# Spatial Distribution of Soil Organic Carbon and Its Influencing Factors in Desert Grasslands of the Hexi Corridor, Northwest China

**DOI:** 10.1371/journal.pone.0094652

**Published:** 2014-04-14

**Authors:** Min Wang, Yongzhong Su, Xiao Yang

**Affiliations:** 1 Linze Inland River Basin Research Station, Chinese Ecosystem Network Research, Cold and Arid Regions Environmental and Engineering Research Institute, Chinese Academy of Sciences, Lanzhou, Gansu, China; 2 University of Chinese Academy of Sciences, Beijing, China; DOE Pacific Northwest National Laboratory, United States of America

## Abstract

Knowledge of the distribution patterns of soil organic carbon (SOC) and factors that influence these patterns is crucial for understanding the carbon cycle. The objectives of this study were to determine the spatial distribution pattern of soil organic carbon density (SOCD) and the controlling factors in arid desert grasslands of northwest China. The above- and belowground biomass and SOCD in 260 soil profiles from 52 sites over 2.7×10^4^ km^2^ were investigated. Combined with a satellite-based dataset of an enhanced vegetation index during 2011–2012 and climatic factors at different sites, the relationships between SOCD and biotic and abiotic factors were identified. The results indicated that the mean SOCD was 1.20 (SD:+/− 0.85), 1.73 (SD:+/− 1.20), and 2.69 (SD:+/− 1.91) kg m^−2^ at soil depths of 0–30 cm, 0–50 cm, and 0–100 cm, respectively, which was smaller than other estimates in temperate grassland, steppe, and desert-grassland ecosystems. The spatial distribution of SOCD gradually decreased from the southeast to the northwest, corresponding to the precipitation gradient. SOCD increased significantly with vegetation biomass, annual precipitation, soil moisture, clay and silt content, and decreased with mean annual temperature and sand content. The correlation between BGB and SOCD was closer than the correlation between AGB and SOCD. Variables could together explain about 69.8%, 74.4%, and 78.9% of total variation in SOCD at 0–30 cm, 0–50 cm, and 0–100 cm, respectively. In addition, we found that mean annual temperature is more important than other abiotic factors in determining SOCD in arid desert grasslands in our study area. The information obtained in this study provides a basis for accurately estimating SOC stocks and assessing carbon (C) sequestration potential in the desert grasslands of northwest China.

## Introduction

Soil plays a crucial role in the global carbon cycle by linking carbon transformation with the pedosphere, biosphere, and atmosphere. Therefore, minor changes in the soil carbon pool will greatly affect the alteration of atmospheric CO_2_ concentration and have potential feedbacks to climate change [1]–[4]. SOC storage in temperate grasslands is heavily studied [5]–[14], while there is little research examining SOC storage in arid regions such as desert-grassland or desert-steppe. Arid regions cover about 47.2% of the earth's land area, with soils containing nearly 241 Pg of soil organic carbon, which is about 40 times more than what was added into the atmosphere through anthropogenic activities [15]. Additionally, soils in these regions are fragile and may experience degradation, desertification, wind erosion, and overgrazing. Small changes in soil conditions can modify the original balance of soil carbon cycle, increase the C loss from soil, and release more greenhouse gases into the atmosphere. Therefore, SOC storage in the desert-grassland ecosystem is a critical component of global C cycle and has a considerable effect on reducing the rate of enrichment of atmospheric CO_2_.

In northwest China, desert-grasslands are widely distributed (near 6.5×10^7^ hm^2^); however, the SOC storage here has not been widely studied. Among the limited estimates of SOC storage in desert-grassland in northwest China [16]–[18], large differences were found, potentially due to different data sources or approaches. Data from the Second National Soil Survey were usually used for these previous estimates [16]–[18], but few soil profiles were sampled from the grasslands in northwest China, and these soil profiles lacked data on bulk density and gravel fractions [19]. Regarding different data approaches, previous studies usually calculated SOC stock using average SOC density (SOCD); however, this approach could be constrained by limited soil profiles and large soil heterogeneity. Accordingly, satellite-based approaches will be useful to scale up site-level observations to regional-scale estimates [19].

SOC storage in grasslands is closely correlated with biological, climatic, and edaphic factors. SOC storage exhibits a balance between C inputs from organic material, and C losses through decomposition and mineralization [20]–[23]. In particular, this balance depends on climatic conditions [18], [24]–[26]. Precipitation and temperature determine the vegetation types, size of plant productivity, and the speed of microbial degradation of soil organic matter. In addition to climate, soil texture plays an important role, which results in an increasing clay content and decreasing C outputs through its stabilizing effect on SOC [5], [26], [27].

The Hexi Corridor, one characteristically arid area in northwest China, with annual precipitation ranging from about 200 mm in the east to less than 50 mm in the west, represents a desert-grassland ecosystem. Desert-grasslands here always contain transition zones between desert and grassland or between desert and oases, and play a crucial role in maintaining a stable ecological environment and productivity. The desert grassland ecosystem in the Hexi Corridor has unique features, such as limited precipitation, low vegetation cover, coarse soil particles, large gravel content, and highly intensified wind erosion. Accordingly, the vegetation composition varies more than other desert-grasslands. For example, desert steppe in Inner Mongolia is mainly composed of gramineous plants and shrubs with deep roots [28], whereas small shrubs or subshrubs with shallow roots dominate the desert grassland in the Hexi Corridor [29], which results in small biomass productivities and low soil C inputs. These characteristics could lead to greater differences in SOC storage compared with other typical grasslands, but few studies have focused on this desert grassland. The working hypothesis for our study was that the spatial distribution of SOC will show clear relationships with unique climate and vegetation biomass, as well as specific soil conditions. Throughout our field investigations, our research objectives were: (1) to identify SOC density and its spatial distribution characteristics; and (2) to analyze the influence of biotic, climatic, and edaphic factors on SOC density and its distribution in an arid desert grassland. Well water conditions and soil particle composition can promote plant growth and soil organic carbon fixation, and high temperature can accelerate the decomposition of soil organic carbon. For these reasons we hypothesized that SOCD would increase with the increase of moisture and soil clay content, and decrease with the rise of temperature.

## Materials and Methods

### Ethics Statements

The location of field studies is not privately-owned or protected in any way, so no specific permission was required. All field studies in the desert-grassland were undertaken with support from Linze Inland River Basin Research Station, Cold and Arid Regions Environmental and Engineering Research Institute, Chinese Academy of Sciences. The field studies did not involve endangered or protected species.

### Study area

The study area is found in the central region of the Hexi Corridor in Gansu province, northwestern China (spanning from 101°42′36″ to 97°45′36″E and 40°31′12″to 38°8′26″N, elevation ranging from 1200 m to 1500 m); the study region has an area of ∼2.7×10^4^ km^2^. The mean annual precipitation varied from 250 mm to 50 mm from the southeast to the northwest, and 70–80% of the rainfall occurs between June and August. The annual mean temperature varies from 5 to 9°C. The main soil types are Calcic-Orthic Aridosols according to Chinese Soil Taxonomy, which is equivalent to the Aridosols and Entisols of the USDA soil taxonomy classification (Group of Chinese Soil Taxonomy, Institute of Soil Science, Chinese Academy of Sciences, 2001). Soil thickness ranges from 0.2 m to 1.5 m, and most soils contain a large amount of gravel (in 0.5–6 cm), especially below the 30 cm soil layer. The vegetation population structure in desert grassland is relatively simple, and the main plant species is composed of small shrubs and sub-shrubs including *Asterothamnus centraliasiaticus*, *Reaumuria songarica*, *Salsola passerina*, *Sympegma regelii* and some ephemeral plant species such as *Suaeda glauca*, *Bassia dasyphylla* and *Artemisia scoparia*. The research areas have been subjected to grazing prohibition since 2000.

### Sampling sites

**Figure 1:**
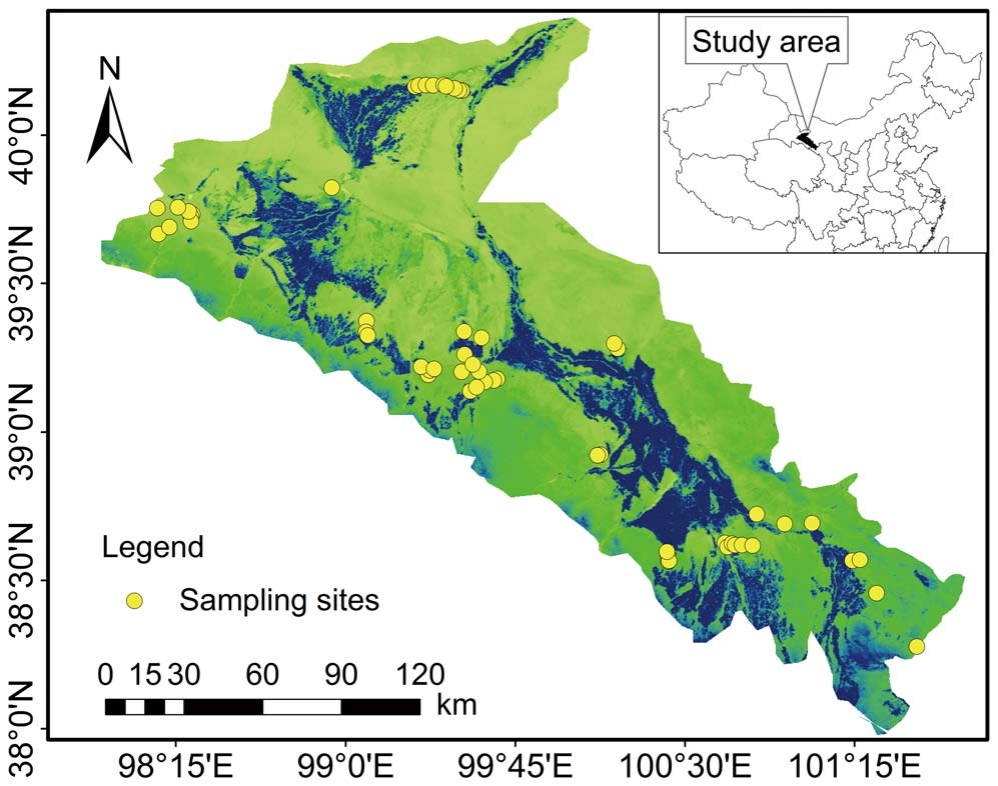
Spatial distribution of sampling sites in the Hexi Corridor.

A total of 52 sites were selected (Figure 1). Each site contains five plots (the area of a square plot is 1 m×1 m for herbaceous plants and small semi-shrubs or 5 m×5 m for shrubs). A total of 260 soil profiles were sampled (i.e., five profiles at each site) in August of 2011–2012. Based on the pre- investigation of vegetation community types in the study area,we set 52 sampling points. These sampling points basically cover the main vegetation communities of the area (*Reaumuria songarica* community, *Salsola passerine* community, *Sympegma regelii* community, and *Asterothamnus centraliasiaticus* community), and can represent the community characteristics, biomass and soil organic carbon contents and other information of desert grassland. On the other hand, we chose areas with no animal dung, no trace of vegetation were eaten, no trace of trampling, and within the barbed wire enclosure as sampling points. That can ensure selected sample sites without human grazing interference.

### Soil sampling, analysis, and biomass survey

At each sampling plot, three soil pits were randomly excavated and mixed into a composite sample at seven depths of 0–5, 5–10, 10–20, 20–30, 30–50, 50–70, and 70–100 cm. Bulk density samples for each depth interval were obtained using a cutting ring (volume of 100 cm^3^). Soil moisture (SM) was measured gravimetrically after 24 h desiccation at 105°C. Bulk density was also calculated as the ratio of the oven-dry soil weight to the cutting ring volume. Soil samples were air-dried, hand-picked to remove plant residues, visible soil organisms, and stones, and weighed. The air-dried samples were then sieved through a screen with 2 mm openings, and gravels (>2 mm) were weighed. The weight percent of gravel to soil was obtained. A proportion of samples that passed through the 2 mm sieve were finely ground to pass through a 0.10 mm sieve and analyzed for soil organic carbon by the K_2_Cr_2_O_7_-H_2_SO_4_ oxidation method developed by Walkley-Black [30]. A subsample was then analyzed for soil texture by the wet sieve method [31].

Aboveground biomass (AGB) and belowground biomass (BGB) were harvested at 260 plots. At sites with herbaceous plants, which were always present at a low plant density (1 m×1 m), we excavated the total plants to obtain aboveground and belowground biomass. At sites with shrubs (5 m×5 m), one or several of the dominant plant species in the plot were chosen according to the crown breadth proportion (large: length × width ≥ 50 cm×50 cm; medium: 20 cm×20 cm≤length × width <50 cm×50 cm, and small: length × width <20 cm×20 cm); the whole plants were then dug out in accordance with the crown breadth survey to estimate above and belowground biomass. In the laboratory, the roots samples were soaked in water and cleaned of residual soil using a 0.5 mm sieve. Biomass samples were oven-dried at 65°C to a constant weight and weighed to the nearest 0.01 g.

### MODIS data and climate information

The MODIS-EVI data used in this study were obtained from the United States Geological Survey at a spatial resolution of 250 m×250 m and 16-day intervals for the period 2011 to 2012 (http://LPDAAC.usgs.gov). Monthly maximum EVI composites were generated using the Maximum Value Composition method proposed by Holben [32] from 2011 to 2012. The EVI data used were the average of monthly EVI during the growing season from July to August.

Climate data, such as mean annual air temperature (MAT) and annual precipitation (AP), were separated from the climate database of the China monthly ground weather dataset during 2011–2012 (http://cdc.cma.gov.cn). These data were spatially interpolated from the records of 25 climatic stations located throughout the Hexi Corridor.

### SOC evaluation

SOC densities for each soil profile at 0–30 cm, 0–50 cm, and 0–100 cm depth intervals were calculated:



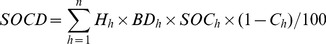
(1)where SOC density (SOCD) in kg m^−2^, soil thickness (*H_h_*) in cm, bulk density (*BD_h_*) in g cm^−3^, SOC (*SOC_h_*) in g kg^−1^, and volume percentage of the fraction >2 mm (*C_h_*) at layer *h* were used.

**Figure 2:**
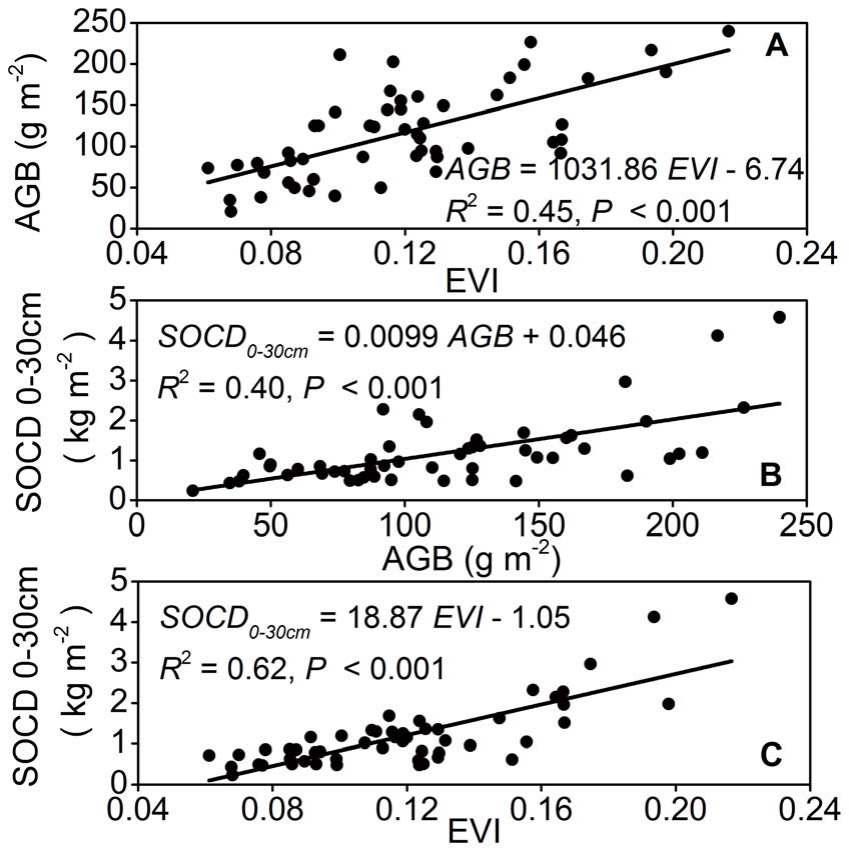
Relationships between above-ground biomass, enhanced vegetation index, and soil organic carbon density at depth interval of 0–30 cm. *Notes*: AGB, above-ground biomass; EVI, enhanced vegetation index; SOCD, soil organic carbon density.

To investigate the spatial distribution of SOCD, we established the relationship between SOCD and MODIS-EVI for three soil depth intervals (Table 1), which was based on the linear relationship between AGB-EVI (Figure 2A) and AGB-SOCD (Figure 2B, and equation for AGB-SOCD_0–50 cm_ and AGB-SOCD_0–100 cm_ was SOCD_0–50 cm_ = 0.014AGB+0.091, *R* ^2^ = 0.41, SOCD_0–100 cm_ = 0.023AGB+0.0039, *R* ^2^ = 0.44, respectively).

**Table 1:** Relationships between soil organic carbon density and enhanced vegetation index at three soil depth intervals (0–30 cm, 0–50 cm, and 0–100 cm).

Soil depth	Equation	R^2^	*P*	RMSE	SSE	F-statistic
0–30 cm	*SOCD*_0–30 cm_ = 18.87 *EVI* - 1.05	0.62	<0.001	0.52	13.97	82.77
0–50 cm	*SOCD*_0–50 cm_ = 25.64 *EVI* - 1.33	0.58	<0.001	0.70	30.76	69.98
0–100 cm	*SOCD*_0–30 cm_ = 37.12 *EVI* - 1.75	0.48	<0.001	1.35	95.32	47.69

*Notes:* RMSE, root-mean-square error; SSE, sum of squares for error.

**Figure 3:**
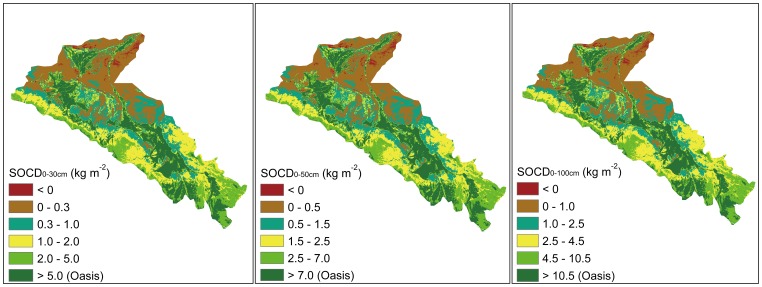
Spatial distributions of soil organic carbon density at different soil layers (0–30 cm, 0–50 cm, and 0–100 cm). *Notes:* SOCD, soil organic carbon density.

Using the regression equations (Table 1), each pixel of EVI was converted to SOCD. We then obtained the spatial distribution of SOCD for different soil layers (Figure 3A–C). The spatial distribution of SOCD was performed in ArcGIS, version 9.3 (ESRI, RedLands, California).

### Statistical analysis

We used simple linear regression to analyze the relationship between dependent and independent variables (AGB-EVI, SOCD-AGB, SOCD-EVI, SOCD-BGB, and SOCD-TB, respectively). Additionally, to evaluate integrative effects of MAT, AP, SM, and soil texture on SOCD, a general linear model (GLM) was employed. Ordinary least squares regression was used to fit these models. Residuals from the linear models were examined for normality (by Shapiro-Wilk test), independence (by Durbin-Watson test), and linearity (by plotting the residuals after fitting linear regression between dependent variable and independent variable) and data met the assumption of normality, independence and linearity. Therefore, linear regressions and GLM models were effective and meaningful for these analyses. Statistical analyses were conducted with R version 3.0.2 package (http://www.R-project.org). We also analyzed the correlations between SOCD and environmental factors (MAT, AP, SM, Silt, Clay, and Sand) using nonlinear regression (by Levenberg-Marquardt and Universal Global Optimization analyses). We constructed exponential equations to describe relationships between SOCD and MAT, AP, SM, soil texture. Nonlinear regression analyses were performed using 1stOpt software, version 1.5 (First Optimization, 7D-Soft High Technology Inc., Xian, China).

## Results

### SOC stocks and spatial distribution

The statistical description of SOCD across 52 sites at soil depths of 0–30 cm, 0–50 cm, and 0–100 cm is shown in Table 2. As shown, SOCD exhibited large variations among the three soil depths, ranging from 0.24–4.58 kg m^−2^ for 30 cm in depth, 0.35–6.32 kg m^−2^ for 50 cm, and 0.59–9.57 kg m^−2^ for 100 cm, respectively. The corresponding average SOC densities were 1.20, 1.73, and 2.69 kg m^−2^, respectively. The SOC content in 0–30 cm interval accounted for almost 45% of total SOC in the top 1 m of soil.

**Table 2:** Statistics of soil organic carbon density at three soil depth intervals (0–30 cm, 0–50 cm, and 0–100 cm).

SOCD	N	Mean (kg m^−2^)	Std.D. (kg m^−2^)	Min (kg m^−2^)	Median (kg m^−2^)	Max (kg m^−2^)
SOCD_0–30 cm_	52	1.20	0.85	0.24	0.99	4.58
SOCD_0–50 cm_	52	1.73	1.20	0.35	1.44	6.32
SOCD_0–100 cm_	52	2.69	1.91	0.59	2.16	9.57

*Notes:* SOCD, soil organic carbon density; N, number of samples; Std.D., standard Deviation.

The density of SOC decreased from the southeast to the northwest (Figure 3A–C), which corresponds to the precipitation gradient.

### Effects of biomass and environmental factors on SOCD

**Figure 4:**
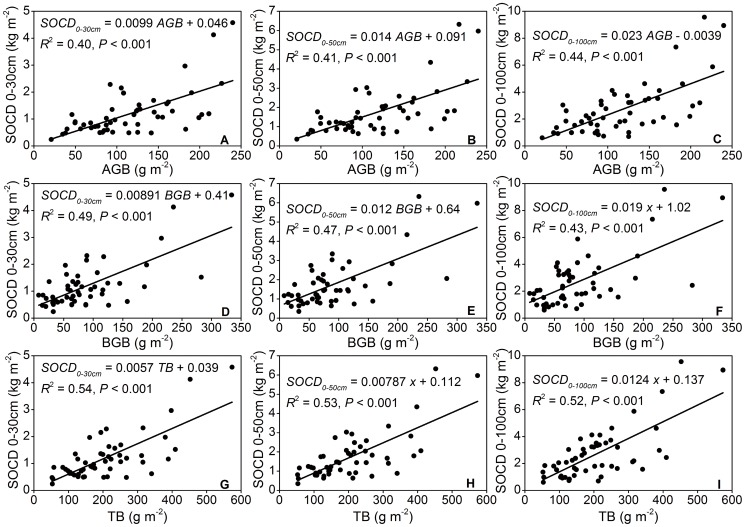
Relationships between soil organic carbon density and biomass at different depth intervals. *Notes*: AGB, above-ground biomass; BGB, below-ground biomass; TB, total biomass; SOCD, soil organic carbon density.

The significant positive relationships between SOCD and AGB, BGB, and TB at different soil depths were characterized by linear functions (Figure 4, *P*<0.001). The *R* ^2^ values of regression functions between TB and SOCD were the highest of three biological variables. The *R* ^2^ values of regression functions between BGB and SOCD were higher than that between AGB and SOCD and thus showed the closer correlation between BGB and SOCD compared with the correlation between AGB and SOCD (Figure 4, A–C for BGB, D–F for AGB) and exhibited the crucial influence of BGB on SOC distribution (Figure 4G–I). The association of SOC content with BGB and TB was closest in the top soil and decreased at deeper intervals (Figure 4D and G). Nevertheless, the relationships between AGB and SOCD exhibited only minimal differences at three soil depths (Figure 4A–C).

**Figure 5:**
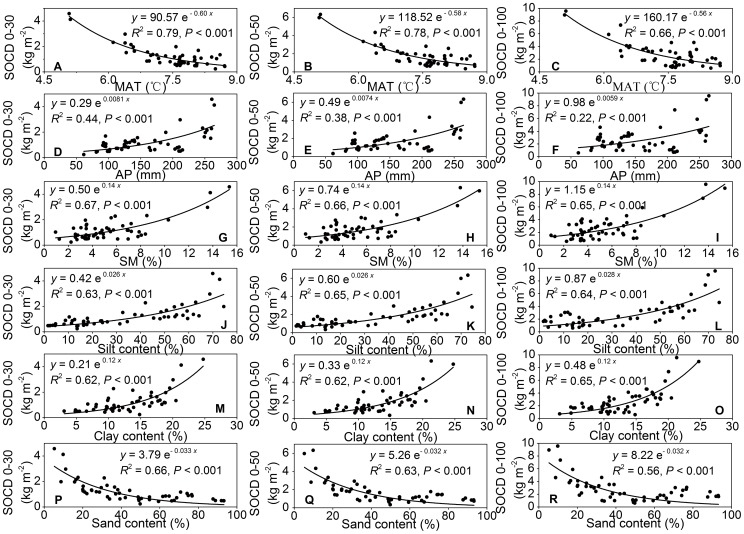
Relationships between soil organic carbon density and environmental factors at different depth intervals. *Notes*: SOCD, soil organic carbon density; MAT, mean annual temperature; AP, annual precipitation; SM, soil moisture.

We established regression relationships between SOCD and various environmental factors, such as MAT, AP, SM, silt, clay, and sand content (Figure 5). In the top 0–30 cm interval, SOCD decreased markedly with MAT (Figure 5A) and sand content (Figure 5P). On the contrary, SOCD increased significantly with an increase SM (Figure 5G). Furthermore, SOCD was positively related with silt (Figure 5J) and clay content (Figure 5M) as well as AP and SM. The regression curves indicated that the closest relationship between SOCD and environmental variables in the top 30 cm soil was with MAT (Figure 5A, *R* ^2^ = 0.79, *P*<0.001), followed by SM (Figure 5G, *R* ^2^ = 0.67, *P*<0.001). Additionally, similar relationships between SOCD and environmental factors were also observed in other soil intervals (Figure 5B, E, H, K, N, and Q for 0–50 cm; Figure 5C, F, I, L, O, and R for 0–100 cm). The *R* ^2^ value of fitted curves for associations of SOCD with MAT, AP, SM, and sand content declined with soil depth, but an increasing trend was found for association of SOCD with silt and clay content (Figure 5).

According to the above-described relationship between SOCD and environmental factors, we chose five variables (MAT, SM, clay, silt, and sand content, and soil moisture as a measure of water availability) to establish a GLM model. The results suggested that environmental factors explained 69.81%, 74.41%, and 78.87% of the overall variation of SOCD at the soil intervals of 0–30 cm, 0–50 cm, and 0–100 cm, respectively (Table 3).

**Table 3:** Integrative effects of mean annual temperature, soil moisture and soil texture (clay, silt, and sand) on soil organic carbon density at three soil depth intervals (0–30 cm, 0–50 cm, and 0–100 cm).

Source	0–30 cm				0–50 cm				0–100 cm			
	df	MS	SS%	P	df	MS	SS%	P	df	MS	SS%	P
MAT	1	4.45***	35.53	−0.47	1	8.70***	33.58	−0.66	1	15.13***	19.31	−0.87
SM	1	3.37***	26.87	0.10	1	6.51***	25.12	0.14	1	18.49***	23.61	0.24
Clay	1	0.33*	2.60	0.026	1	1.22**	4.69	0.051	1	7.04***	8.99	0.12
Silt	1	0.52*	4.14	0.013	1	1.89**	7.28	0.024	1	10.46***	13.36	0.057
Sand	1	0.08	0.67	0.004	1	0.97*	3.74	0.014	1	10.65***	13.60	0.047
Residuals	46	0.08	30.19		46	0.14	25.59		46	0.36***	21.13	
Intercept				3.22				3.78				2.25

*Notes*:

^***^P<0.001;

^**^P<0.01;

^*^P<0.05.

df, degree of freedom; MS, mean squares; SS%, proportion of variances explained by the variable; P, parameters of best-fitted GLM equations; MAT, mean annual temperature; SM, soil moisture.

The results were obtained from general linear model analysis.

MAT was the most important parameter for SOCD at 0–30 cm and 0–50 cm (and accounted for 35.53% and 33.58% of variation), whereas SM was the most important parameter for SOCD at 0–100 cm (where it accounted for 23.61% of variation). The proportion of variances explained by both factors decreased with an increase of soil depth. Soil texture variables (clay, silt, and sand content) explained 7.41%, 15.71%, and 35.95% of the variance at 0–30 cm, 0–50 cm, and 0–100 cm, respectively. In contrast to MAT and SM, the proportion of variance explained by soil texture markedly increased along with soil depth. The proportion of variance explained by sand content increased more rapidly than those explained by the other two variables, and instead silt content become the most important textural variable at soil depth of 0–100 cm (accounted for 13.60% of variation).

## Discussion

### SOC storage estimation

We summarized previous estimations on SOC storage on different vegetation types at global and regional scales in Table 4. In this study, the mean SOCD of 260 soil profiles at a soil depth of 0–100 cm in the desert grassland of the Hexi Corridor was 2.69 kg m^−2^. Our results were generally lower than the global mean SOCD (10.8 kg m^−2^), the average SOCD in China (7.8 kg m^−2^), and other records based on vegetation types (temperate desert, steppe, and grassland). This difference is most likely due to differences in climate and soil conditions, which play a critical role in determining vegetation types and biomass productivity. The drier local climate, together with a greater gravel content and thinner soil layer thickness compared to other temperate steppe, grassland, and desert-grassland regions lead to lower vegetation production and limited SOC inputs [29].

**Table 4:** Comparisons of soil organic carbon density in desert grasslands in the Hexi Corridor with previous estimates.

Research types	Soil organic carbon density (kg m^−2^)				Reference
	0–30 (cm)	0–50 (cm)	0–100 (cm)	Actual depth (cm)	
Global mean	−	−	10.8	−	[23]
Global cool temperate desert	−	−	9.7	−	
Global cool temperate steppe	−	−	13.3	−	
Global temperate grassland	−	−	11.7	−	[26]
Global desert	−	−	6.2	−	
Average of China	3.7	−	7.8	−	[18]
Temperate grassland of Inner Mongolia	4	5.19	6.68	−	[28]
Desert steppe of Inner Mongolia	2.3	3.1	4.01	−	
				−	
Temperate typical steppe	−	−	12.3	−	[40]
Temperate deserted steppe	−	−	8.7	−	
Temperate desert	−	−	6.2	−	
Temperate steppe-desert	−	−	8	−	[10]
Temperate desert-steppe	−	−	8.7	−	
Temperate desert	−	−	6.2	−	
Desert	−	−	4.39	−	[41]
Desert steppe	−	−	7.09	−	
Temperate typical steppe	−	−	−	8	[16]
Temperate deserted steppe	−	−	−	2.8	
Temperate desert	−	−	−	2.2	
Desert grasslands in the Hexi Corridor	1.2	1.73	2.69	−	This study

*Notes*: “−” mean not measured.

### Relationship between SOCD and biomass

SOC concentrations are closely linked to biotic processes, such as biomass production, decomposition, and the placement of aboveground litter and root litter in and on the soil [33]. Through regression analyses, we detected a higher coefficient of *R* ^2^ for the linear functions between SOCD and BGB compared with the association between SOCD and AGB at the three soil depth intervals. This finding indicated that BGB was most likely the main resource of C inputs and a dominant biological factor on the determination of SOCD. Belowground roots can provide abundant and stable organic material into soil and enhance SOC density. However, due to intense wind erosion in our research area, large amounts of litter fall were blown away by wind, which resulted in only a small amount of aboveground organic matter entering the soil.

Both BGB and TB exhibited the highest correlation in the top 30 cm soil, and this correlation decreased at deeper intervals likely due to high organic matter inputs at surface soil [26], [34]. Large gravel content at depths below 30 cm make it hard for roots to extend to deep soil layers, so most of root biomass is concentrated in the upper 0–30 cm soil interval (almost 97%) [29]. According to the shallow distribution of roots, the majority of C inputs from roots are concentrated at soil depths of 0–30 cm; additionally, the topsoil is the first layer that directly receives C inputs from aboveground biomass.

### Relationship between SOCD and climate and soil environmental factors

Under natural conditions, the distribution of SOC was controlled by climate, vegetation, parent material, and soil texture [3], [5], [23], [26], [35]. Our studies observed SOC distribution was positively associated with precipitation and clay content and negatively correlated with temperature and sand content and indicated that the strength of relationships between SOCD and environmental variables decreased with the increase of soil depth.

As shown in Figure 5, SOCD decreased markedly with MAT, likely due to the accelerated mineralization of soil organic matter with increasing temperature [3], [24], [26]. On the other hand, increasing temperature in arid regions results in a considerable decline in water use efficiency by increasing evapotranspiration can lead to low biomass production and low SOC density [15]. Additionally, the effect of MAT on SOCD in each soil layer was stronger than that for AP and edaphic factors. This result underlines the importance of MAT as a predicator for the SOC density in desert grasslands.

In arid ecosystems, precipitation and soil moisture constrain plant production and decomposition [26]. Our results revealed a substantial increase in the SOCD in desert grasslands with an increase in AP and SM. These results imply that water availability is a powerful parameter for assessment of SOCD. Water is the limiting factor for plant production in desert grasslands, as a small increase of water could significantly stimulate bio-productivity and thus contribute to the accumulation of SOCD [26], [36]. Meanwhile, higher precipitation and soil moisture will affect SOC sequestration mainly through higher soil acidity and lower base saturation at the exchange sites, which would reduce the litter decomposition rate. In addition to the abovementioned results, the relationship between SOCD and SM was stronger than that between SOCD and AP. Precipitation in arid desert-grassland has difficulty entering the soil through infiltration due to low vegetation coverage, less litter content, and coarse soil texture, and consequently, soil moisture can represent the actual water content and play a more important role in model construction between SOCD and environmental factors.

In our study, we observed that the increased SOCD was positively correlated with the accumulation of silt and clay content but negatively correlated with sand content. Finely textured soils with appropriate clay and silt content can increase physical and hydrological protection of SOC by inhibiting decomposition through stabilizing SOC, and increasing residence time to decrease C leaching [3], [5], [26], [37], [38]. Moreover, an increase in clay and silt content could enhance the formation of aggregates, which have two advantages for improving SOC content: (1) by improving the soil quality and capacity to efficiently retain water [3], [5], [39] and (2) by mitigating wind erosion [27]. These two advantages could stimulate plant productivity and thus result in additional C inputs.

The GLM analysis suggested that climate factors were more important in determining the distribution of SOC than soil texture, but the effect decreased gradually with an increase in soil depth. In contrast, the impact of soil texture on SOC distribution in surface soils was not obvious but increased alongside soil depth, which indicates that soil texture plays a critical role in SOC distribution at deeper soil layers. Among all climatic and edaphic parameters, MAT had the highest contribution to explain the distribution of SOC in surface soils (0–30 cm and 0–50 cm). Considering the vast majority of SOC accumulates in the surface soil [26], MAT plays a decisive role in the spatial distribution of SOC in the arid desert- grassland ecosystem in the Hexi Corridor. That may be because biomass production is strictly limited by water in arid desert- grasslands, and the rise of temperature can promote the decomposition of SOC, and conversely promote the accumulation of SOC.
